# Differentially expressed gene networks, biomarkers, long noncoding RNAs, and shared responses with cocaine identified in the midbrains of human opioid abusers

**DOI:** 10.1038/s41598-018-38209-8

**Published:** 2019-02-07

**Authors:** Manal H. Saad, Matthew Rumschlag, Michael H. Guerra, Candace L. Savonen, Alaina M. Jaster, Philip D. Olson, Adnan Alazizi, Francesca Luca, Roger Pique-Regi, Carl J. Schmidt, Michael J. Bannon

**Affiliations:** 1 Wayne State University School of Medicine, Department of Pharmacology, Detroit, MI 48201 USA; 2 Wayne State University School of Medicine, Center for Molecular Medicine & Genetics, Detroit, MI 48201 USA; 3 Wayne State University School of Medicine, Department of Obstetrics and Gynecology, Detroit, MI 48201 USA; 4 University of Michigan School of Medicine, Department of Pathology, Detroit, MI 48109 USA

## Abstract

Opioid abuse is now the most common cause of accidental death in the US. Although opioids and most other drugs of abuse acutely increase signaling mediated by midbrain dopamine (DA)-synthesizing neurons, little is known about long-lasting changes in DA cells that may contribute to continued opioid abuse, craving, and relapse. A better understanding of the molecular and cellular bases of opioid abuse could lead to advancements in therapeutics. This study comprises, to our knowledge, the first unbiased examination of genome-wide changes in midbrain gene expression associated with human opioid abuse. Our analyses identified differentially expressed genes and distinct gene networks associated with opioid abuse, specific genes with predictive capability for subject assignment to the opioid abuse cohort, and genes most similarly affected in chronic opioid and cocaine abusers. We also identified differentially expressed long noncoding RNAs capable of regulating known drug-responsive protein-coding genes. Opioid-regulated genes identified in this study warrant further investigation as potential biomarkers and/or therapeutic targets for human substance abuse.

## Introduction

Drug-related deaths, the majority of which involve opioid use, now exceed all other causes of accidental death in the U.S. The past several decades have been characterized by parallel increases in prescription opioid sales, opioid treatment admissions, and prescription opioid overdose deaths, followed by a resurgence in heroin abuse and deaths and, most recently, a steep rise in opioid deaths involving fentanyl or fentanyl analogs^1,2^. The frequent co-abuse of opioids and non-opioid drugs such as cocaine^3^ is another complexity of the current opioid epidemic. A better understanding of the molecular underpinnings of substance abuse could lead to advancements in terms of therapeutic interventions for this disorder. Although animal models are essential for implementation of mechanistic studies, human-specific differences in gene expression, neuroanatomy, and patterns of drug use argue that the direct assessment of human postmortem brain is also critically important^4^.

Drugs of abuse share the property of acutely increasing the signaling of dopamine (DA)-synthesizing midbrain neurons to a number of forebrain targets^5,6^. With continued drug use, neuroadaptations such as drug dependence, craving, and relapse are thought to arise from persistent changes in gene expression^5,6^. While such changes have been identified in human forebrain targets of DA signaling (e.g. nucleus accumbens, prefrontal cortex)^4,7–9^, little is known about any changes arising within midbrain human DA neurons themselves, despite their critical role in the circuitry of addiction.

The current investigation represents, to our knowledge, the first unbiased examination of genome-wide changes in midbrain gene expression associated with human opioid abuse. Analyses revealed differentially expressed genes and gene networks associated with opioid abuse, including those genes more predictive of subject assignment to the correct (drug-free or opioid-abusing) cohort, and those most similarly affected by opioid and cocaine use. Based on these data, further study seems warranted to determine the potential of these genes as biomarkers and/or therapeutic targets for substance abuse.

## Results and Discussion

### Identification of differentially expressed genes and distinct gene co-expression networks in ventral midbrain associated with opioid abuse

**Figure 1:**
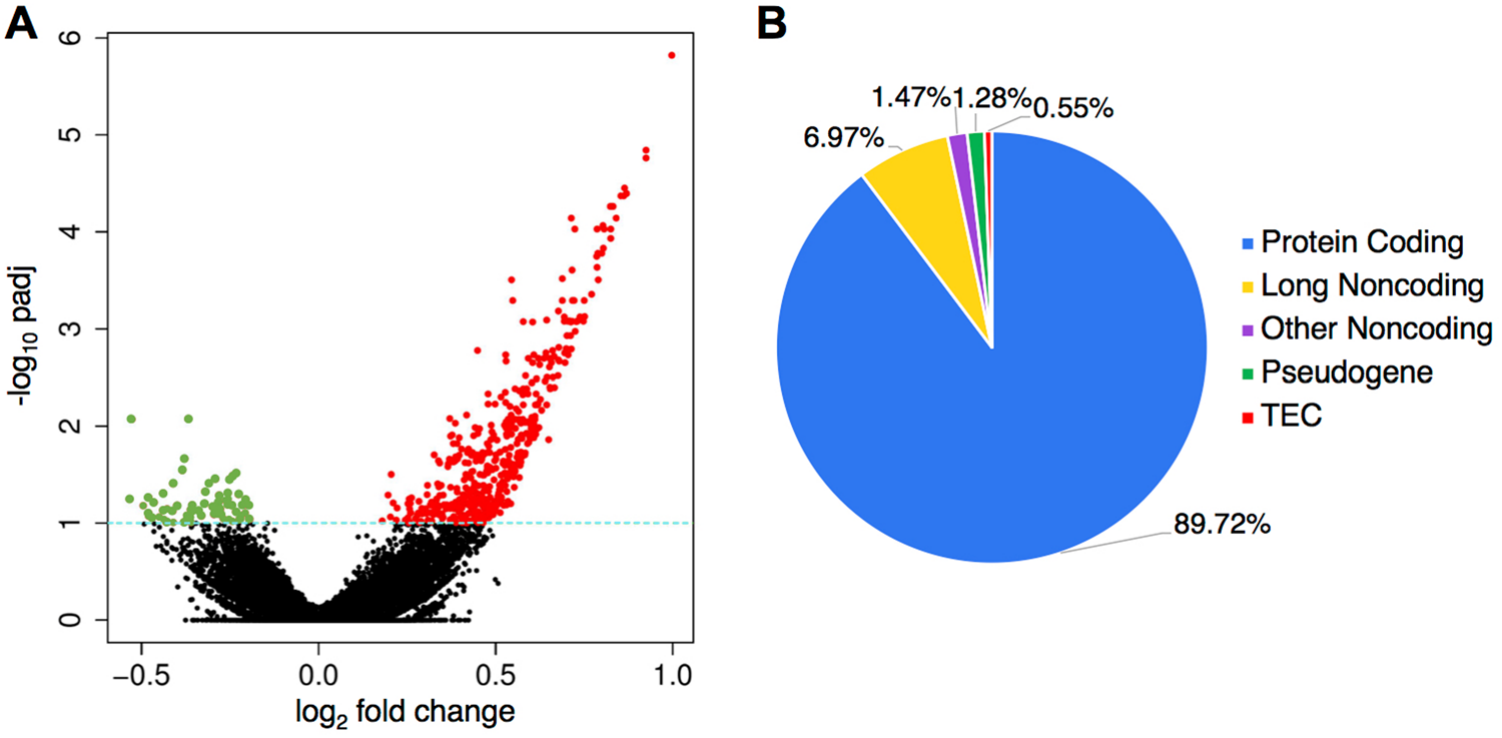
Genes differentially expressed in the midbrain of opioid abusers. Differentially expressed genes were determined using DESeq2 as described in Methods (**A**). Fold change plotted against adjusted p-value (padj < 0.1 considered nominally significant). Note that the preponderance of differentially expressed genes were up-regulated (red), with a smaller number down-regulated (green) (**B**). Biotype of genes differentially expressed. Protein-coding genes encompass the vast majority of these genes; the second largest group by class was long noncoding RNAs. TEC, to be experimentally confirmed.

To ascertain changes in gene expression associated with opioid abuse, we performed high-throughput RNA-sequencing on postmortem ventral midbrain specimens from chronic opioid users (N = 30) and drug-free control subjects (N = 20), as described in the Methods. The cohorts were well-matched in terms of demographic characteristics and measures of sample quality (Table 1). For more detailed information on the characteristics of individual subjects, see Supplementary Table S1. Using DESeq2^10^, after regressing out covariates (shown in Supplementary Fig. S1), we identified 545 (of 36,283) genes as being differentially expressed (p-adj < 0.1), the majority of which were protein-coding (89.7%) and up-regulated (87.7%) (Fig. 1). A complete list of differentially expressed genes can be found in Supplementary Table S2.

**Table 1:** Summary of subject demographics and specimen quality.

	Control Subjects (n = 20)	Opioid Abuse Subjects (n = 30)
Age	50.4 ± 0.93	51.5 ± 1.03
Race/Sex		
Black Male	14 (70%)	22 (73%)
White Male	6 (30%)	8 (27%)
Brain pH	6.60 ± 0.03	6.53 ± 0.03
RIN	7.39 ± 0.11	7.25 ± 0.09

Abbreviation: RIN, RNA integrity number.

**Figure 2:**
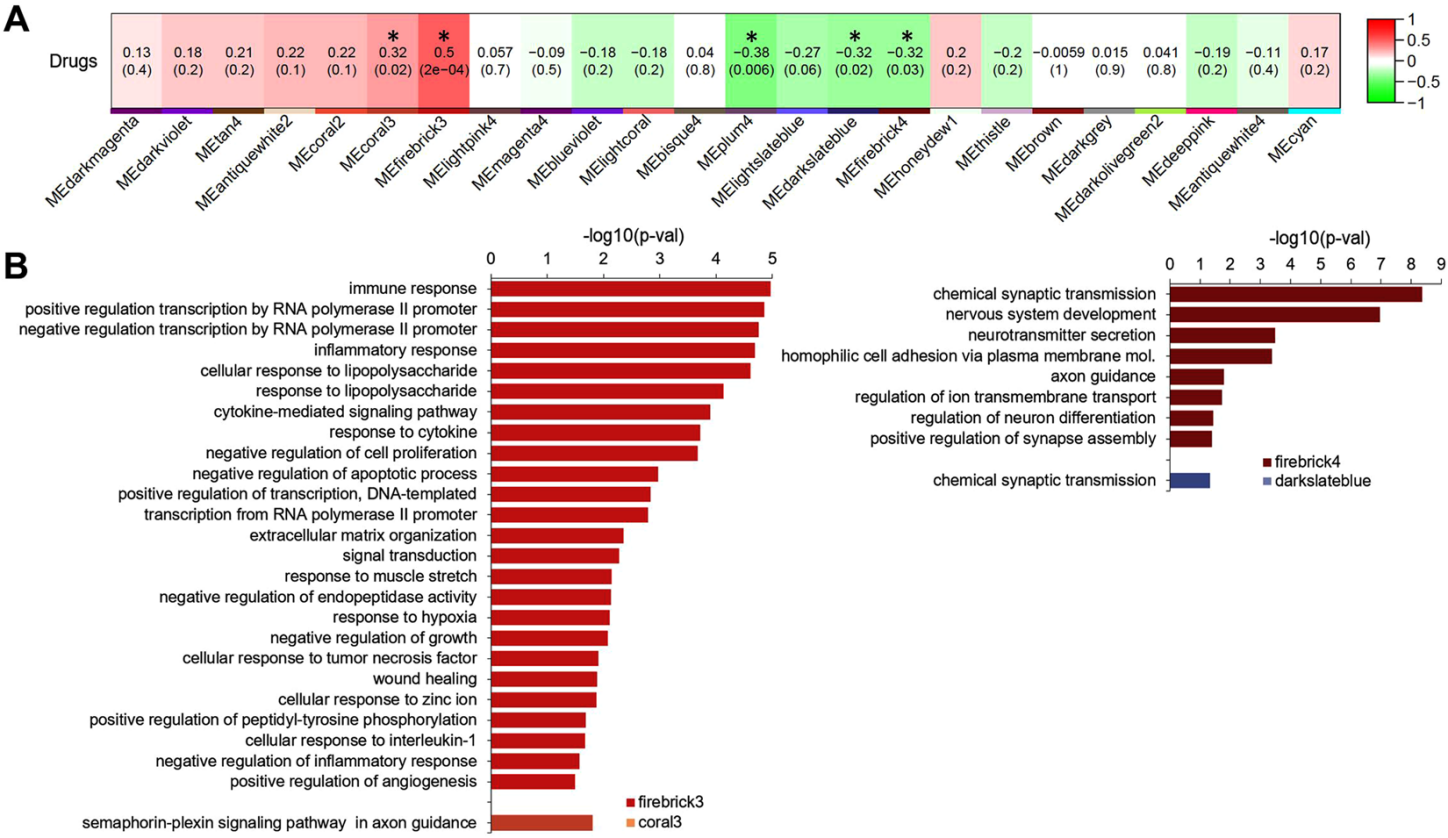
Identification of gene modules dysregulated in opioid users and their associated biological processes (**A**). Pearson correlations were performed between WGCNA module eigengenes and opioid abuse phenotype, as described in Methods. Cells are color-coded using color gradients of red (positive correlations) or green (negative correlations). Five modules were significantly changed with opioid use (p values shown within modules) (**B**). Biological processes enriched in the 4 largest opioid-responsive modules, as determined by DAVID GO analysis as described in Methods (Benjamini-corrected p values shown).

We next applied weighted gene correlation network analysis (WGCNA)^11^ in order to cluster genes into modules based on the degree of correlation of expression patterns. Using this approach, after regressing out covariates we identified 5 of 25 modules as being significantly dysregulated in opioid users (Fig. 2A; for details of clustering of module eigengenes, see Supplementary Fig. S2). As expected, genes differentially expressed in opioid abusers were significantly enriched in these opioid-associated modules (odd ratio 13.77; adjusted p < 2.2E-16, Fisher’s Exact Test).

Gene ontology analysis^12^ revealed that the two up-regulated modules (firebrick3 with 525 genes and coral3 with 25 genes; Fig. 2B) were highly enriched for genes involved in biological processes that included immune, inflammatory, cytokine, and hypoxia responses, semiphorin-plexin signaling, and the regulation of transcription. It was noteworthy that the firebrick3 module included numerous AP-1-related transcription factors (e.g. *FOS, FOSL1, FOSL2, JUN, JUNB, ATF3*), and was highly enriched (p = 1.21E-07) for genes possessing AP-1 binding sites, i.e. the likely target genes for these transcription factors. Similarly, firebrick3 included NFKB-related genes (*NFKB2, NFKBIA*) and was highly enriched (p = 6.12E-11) for genes with NFKB binding sites. These data indicate that FOS/JUN- and NFKB-related transcription factors most likely mediate much of the up-regulation in gene expression that is seen in opioid users’ midbrain (Fig. 1).

On the other hand, the two largest down-regulated modules in opioid users’ midbrains (firebrick4 with 1054 genes, darkslateblue with 767 genes; Fig. 2B) were highly enriched for genes that mediate chemical synaptic transmission and neurotransmitter secretion, nervous system development and differentiation, cell adhesion and axon guidance, ion transport, and synapse assembly. These modules included fewer transcriptional regulators than firebrick3, and were not enriched for target genes containing cognate response elements. The mu opioid receptor gene *OPRM1* was located in these down-regulated modules, as were numerous genes encoding general synaptic machinery, and biosynthetic and/or receptor proteins for the neurotransmitters GABA, glutamate, serotonin, and acetylcholine. Many individual genes within these large modules showed non-significant tendencies toward down-regulation. In a similar vein, genes that specify DA neuron phenotype, whether found in firebrick4 (e.g. *NR4A2, DDC, SNCA, SLC18A2*) or in other modules (e.g. *TH, SLC6A3, DRD2*), were not significantly down-regulated in opioid users. The modular associations of all differentially expressed genes are provided in Supplementary Table S2.

### Predictive capability of individual transcripts for subject assignment to opioid abuse or control groups

**Figure 3:**
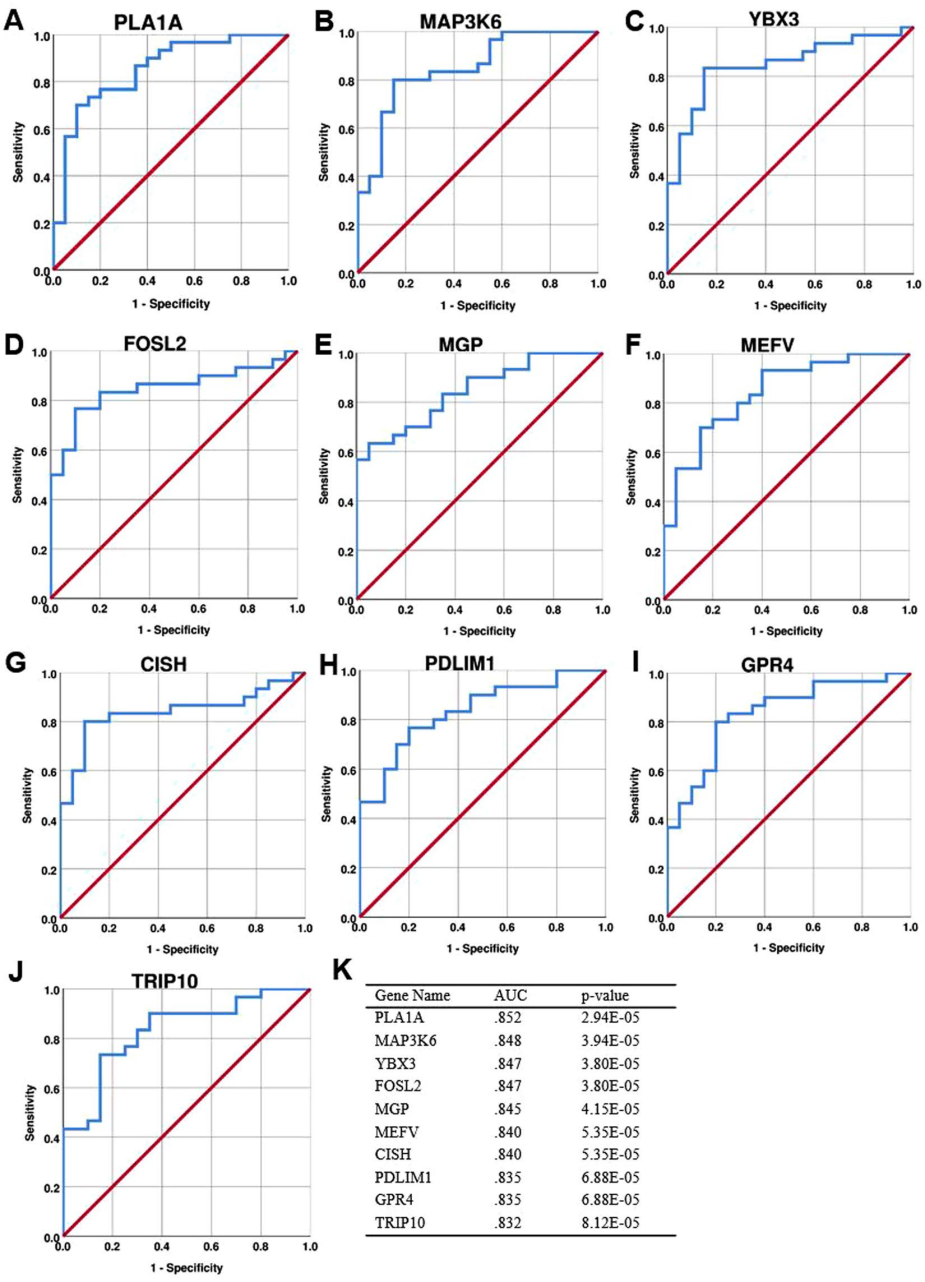
Top ten transcripts in terms of diagnostic performance for subject assignment to correct cohort (**A**–**J**). ROC curves showing the diagnostic performance of the top ten up-regulated genes (by AUC values). The red diagonal line represents chance-level performance and is included as a reference (**K**). AUC values and asymptotic p-values for the genes shown in (**A**–**J**).

We used receiver operating characteristic (ROC) curve analysis^13^ to assess the predictive capability (i.e. sensitivity, specificity, and statistical significance) of individual transcripts to serve as biomarkers for correct subject assignment to opioid use versus control groups. Figure 3 shows data for the top 10 ROC-significant, up-regulated genes, all of which were part of the firebrick3 module (ROC AUC data for all differentially expressed genes can be found in Supplementary Table S2). Only one of these genes, *FOSL2* (encoding the transcription factor FRA-2; Fig. 3D), has been previously implicated in substance abuse on the basis of genetic and neurochemical studies^14,15^. Another highly ROC-significant gene is *GPR4* (Fig. 3I), which encodes a neuronal proton receptor that mediates regulation of CO_2_-stimulated breathing^16^. Because opioids acutely depress the respiratory drive to CO_2_ but significant tolerance develops to this effect, *GPR4* up-regulation may reflect an important compensatory response to chronic opioid use. Other ROC-significant genes include *TRIP10* (encoding CIP4; Fig. 3J), which regulates synaptic strength via AMPA receptor endocytosis^17^, and *YBX3* (encoding Y-box protein 3; Fig. 3C), which broadly regulates gene expression via RNA binding and other mechanisms^18^. The remaining top ROC-significant genes have been implicated in immune, inflammatory, or ischemic responses primarily in non-neural tissue, but potential roles related to drug abuse or broader CNS function have not been elucidated previously. Boxplots for these top ROC-significant genes are shown in Supplementary Fig. S3. The present data suggest that these genes should be further assessed in larger, independent cohorts in terms of their potential as biomarkers for opioid abuse.

### Potential involvement of long noncoding RNAs in the response to opioids

**Figure 4:**
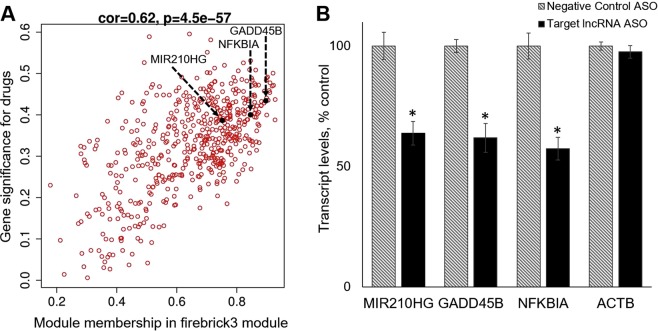
LncRNA *MIR210HG* regulates expression of downstream target genes *GADD45B* and *NFKBIA* (**A**). The relationship between gene significance (a measure of the correlation between a given gene and the opioid variable) and module membership (the relationship between a given gene and the module eigengene) is shown for genes in the firebrick3 module. Overall correlation and significance are noted at the top of the panel. Several genes with highly correlated co-expression in human midbrain, which were subsequently investigated in SKNAS-G cells (panel B), are indicated (**B**). DAergic SNKAS-G cells were treated for 24 hr with ASO directed against *MIR210HG*, or a negative control ASO. Selective knockdown of *MIR210HG* resulted in corresponding, selective reductions in expression of *GADD45B* and *NFKBIA*, but not a beta actin negative control (*ACTB*). The results of two independent experiments were combined (total N = 8 samples per group). *p < 0.0005.

Although the majority of genes within the opioid-associated modules were protein-coding in nature, we noted the presence of a number of long noncoding RNAs (lncRNAs; Supplementary Table S2) that exhibited strong modular membership and drug-responsiveness (i.e. they were module hub genes). The functionality of most putative lncRNAs has not been investigated, but some lncRNAs have been shown, in other systems, to regulate expression of downstream protein-coding gene targets^19^. We investigated this possibility for several of our differentially expressed lncRNAs through experimental manipulation of their expression in SKNAS-G cells, a previously characterized^20^ cell model of human DA neurons (as shown in Fig. 4 and Supplementary Fig. S4). *MIR210HG*, an lncRNA hub gene in firebrick3 (Fig. 4A), has been previously identified in several models systems as a highly hypoxia- and ischemia-responsive gene^21,22^, in keeping with the general processes attributed to the firebrick3 module (Fig. 2). Acute knockdown in SKNAS-G cells of *MIR210HG* levels elicited parallel decreases in the levels of *GADD45B* and *NFKBIA* (Fig. 4B), two other firebrick3 hub genes whose expression was also significantly correlated (1E-06 and 6E-07, respectively) with *MIR210HG* in human midbrain (Fig. 4A).

*GADD45B*, has widespread effects on neural gene expression via DNA demethylation^23–25^, and *NFKBIA* critically regulates neural NKFB-mediated transcription^26^; both have been implicated in drug abuse^26–28^. It is noteworthy that *GADD45B* and *NFKBIA* are robustly up-regulated in the midbrains of both human opioid abusers (present study) and cocaine abusers^29,30^. Importantly, we found that *MIR210HG* was selectively expressed within human DA neurons (Supplementary Fig. S5), as are *GADD45B* and *NFKBIA*^29^. The present data suggest that the lncRNA *MIR210HG* may be an important upstream mediator of a shared transcriptional response of DA neurons to chronic opioid or cocaine use.

Parallel experiments were conducted on another lncRNA hub gene in firebrick3, *LINC00963*, previously described in other contexts as a positive regulator of cell proliferation and motility^31^. Acute knockdown of *LINC00963* in SKNAS-G cells (Supplementary Fig. S4) led to a significant down-regulation of expression of *GPT2*, a gene encoding a mitochondrial enzyme which, when disrupted *in vivo*, causes decreases in brain size and synapse formation^32^. Different lncRNA hub genes in firebrick3 may thus serve as modulators of distinct biological functions subsumed in this opioid-responsive gene module.

### Comparison of present data with gene expression changes previously seen in human cocaine abusers

Beyond the changes in *GADD45B* and *NFKBIA* expression mentioned above, we noted that >11% of the genes differentially expressed in opioid abusers’ midbrains were previously identified as differentially expressed in cocaine abusers’ midbrains^29^, despite differences in study methodologies, subject demographics, and cellular sites of drug action. Two-thirds of these shared differentially expressed genes (listed in Supplementary Table S2) fell into the opioid-responsive, up-regulated firebrick3 module, suggesting a significant overlap in terms of the effects of opioids and cocaine on transcriptional regulation and immune/inflammatory responses in human midbrain. This may be an important observation for the development of potential therapeutic interventions for substance abuse.

On the other hand, although the genes down-regulated in opioid users (Supplementary Table S2) and cocaine users^29^ were both associated with broad neural-related gene ontology categories, there seemed to be little overlap in terms of the specific genes affected. Furthermore, the present data and a previous report^33^ suggest that chronic opioid abuse has relatively modest effects on the expression of genes that specify the DA neuron phenotype, whereas these genes are quite robustly down-regulated in human cocaine abusers^29,30,34–36^. Presumably such differences relate to the distinct cellular sites and mechanisms of action by which opioids and cocaine exert effects in the midbrain, but the implications of this pathophysiological distinction are not fully understood.

### Limitations of the study

A number of potential limitations inherent in postmortem studies of substance abusers warrant mention. The opioid abuse subjects in this study had both a documented history of chronic illicit drug use *and* a positive opioid toxicology at death, rendering it a challenge to parse out acute versus chronic drug effects on the observed changes in gene expression. Further, subjects’ histories of drug preference, and possible co-morbidity for psychiatric disorders, are difficult to ascertain^4^. These limitations notwithstanding, in previous studies we’ve found that differential gene expression in substance abusers’ brain seems to be largely independent of either the immediate cause of death or drug levels at death^30^, and that gene expression profiles of heroin abusers and cocaine abusers are highly distinctive^7^.

The admixture of RNA from multiple cell types found within human ventral midbrain may preclude the identification of very low abundance transcripts, and places some constraints on interpretation of the data. WGCNA-based bioinformatic analysis suggests that whereas the down-regulated modules are significantly enriched for neuronal genes, the up-regulated module firebrick3 is enriched for glial (primarily microglial) markers (Supplementary Fig. S6). On the other hand, direct experimental evidence shows that at least a subset of the most robustly differentially expressed firebrick3 genes is expressed predominantly within DA neurons rather than glia (e.g. *GADD45B, NFKBIA, FOS, EGR1, CCL2, and SERPINA3*^29^; *MIR210HG* (Supplementary Fig. S5)). It is plausible that this module encompasses genes involved in microglia-DA neuron interactions elicited in response to opioids and other drugs of abuse. Unequivocal determination of the cellular loci of midbrain expression of all opioid-responsive genes will require RNA-seq analysis of single cells or sorted cell types. Parallel studies of other brain regions involved in the circuitry of substance use are also needed to better understand this complex and devastating disorder.

## Conclusions

This study represents, to our knowledge, the first unbiased examination of genome-wide changes in midbrain gene expression associated with human opioid abuse. The results indicate that human opioid abuse elicits wide-ranging transcriptional changes in the midbrain which could be categorized into a number of interrelated gene networks and signaling pathways. Two large drug-responsive gene modules (firebrick4 and darkslateblue) associated with the control of synaptic transmission and other neural functions were characterized by relatively modest down-regulation of expression in opioid users. The most striking finding in the present study was the identification of a large drug-responsive module (firebrick3) associated with immune/inflammatory/hypoxic responses and the broad regulation of transcription. Firebrick3 contained a large proportion of the differentially expressed, up-regulated genes seen in opioid abusers, including the genes most predictive (by ROC analysis) of correct subject assignment to the opioid-using cohort. Bioinformatic analyses indicated that numerous transcription factors within firebrick3 may broadly induce expression of downstream target genes in the same module through interactions with cognate binding sites. In addition, direct experimental evidence of downstream gene regulation by specific lncRNAs was obtained. A number of the genes up-regulated in human opioid abusers have been previously found up-regulated in cocaine abusers as well. Given the high incidence of polydrug abuse, these genes in particular warrant further study, to assess their potential as biomarkers and/or therapeutic targets for substance abuse.

## Methods

### Case selection

Human midbrain specimens were collected during routine autopsy and de-identified specimens were characterized as described previously^7,8,29,30,34–36^. The use of de-identified cadaver specimens is not defined as human subjects research and exempt from regulation 45 CFR pt 46 (NIH SF424 guide Part II: Human Subjects). Briefly, cause of death was determined by forensic pathologists following medico-legal investigations evaluating the circumstances of death including medical records, police reports, autopsy results, and extensive toxicological data. Case inclusion in the opioid abuse group (n = 30) was based on a documented history of opioid abuse, toxicology report positive for opioids, and forensic determination of opioids as cause of death. Opioid abusers with a positive toxicology for non-opioid drugs of abuse (e.g. alcohol, cannabinoids, anxiolytics, barbiturates) were excluded from the study, with the exception of inclusion of a subset of cases positive for opioids plus cocaine, as this reflects the drug most commonly co-abused with opioids^3,37^. Cases in the control group (n = 20) had no documented history of drug abuse, and tested negative for opiates, cocaine, and other drugs of abuse or CNS medications. Causes of death for control cases were cardiovascular events or gunshot wounds. Exclusion criteria for either group included a known history of neurological or psychiatric disorder, death by suicide, evidence of neuropathology, debilitating chronic illness, estimated postmortem interval >20 h, or biochemical evidence of poor tissue sample quality or prolonged perimortem agonal state^38,39^ (i.e. brain pH < 6.2 or RNA integrity number [RIN] < 6.0). To reduce variance unrelated to drug abuse, the two groups were matched in terms of gender, race, age, brain pH, and RIN. Table 1 shows a summary of these demographic and sample quality characteristics; complete information for each case is provided in Supplementary Table S1.

### Sample processing and RNA-seq analysis

Sample processing has previously been described in detail^7,8,29,30,34–36^. Briefly, post-mortem samples encompassing the entire ventral midbrain (corresponding to atlas plates 51–56^40^) were fresh-frozen upon collection at autopsy, cryosectioned, and DA cell-enriched regions finely dissected and pooled for each subject. RNA was isolated via homogenization and Trizol extraction, DNase-treated and purified with Qiagen RNeasy mini-kits (Qiagen, USA), and quantified and assessed for integrity using a Bioanalyzer 2000 (Agilent, USA). Isolated RNA was used to prepare RNA-seq libraries using Illumina TruSeq Stranded mRNA High Throughput Sample Preparation Kits (Illumina, USA) following the manufacturer’s protocol. The resulting cDNA library was sequenced on the Illumina NextSeq500 to generate 150 bp paired end reads. We collected a total of ~1.48 billion paired end reads, with an average of 29.6 million raw reads per sample (after removal of a single sample with extremely low [7560] raw read counts). Sequencing reads were aligned to the UCSC Human Genome Browser hg38 assembly with STAR-2.5.0b^41^. Using SAMtools^42^, outputted SAM files were then converted to BAM files. Also using SAMtools, transcript reads were sorted based on aligned chromosomal locations, and resulting BAM files were merged into a single BAM file per sample. Furthermore, quality control was performed through SAMtools to remove multi-mapped reads (q = 10) and PCR duplicates. This resulted in 921 million uniquely mapped reads and 730 million reads after removing PCR duplicates – an average sequencing depth of 14.6 million reads per sample after removing PCR duplicates. HTSeq was used to determine the read counts per gene based on Ensembl gene-level annotations from Gencode release 24 (GRCh38.p5)^43^. These counts were established using the HTSeq gene union mode. The reads after removal of PCR duplicates, and after HTSeq gene alignment, for each subject are listed in Supplementary Table S3. The final un-normalized counts were assembled into a count-matrix through customized R-scripts, and this served as input for DESeq2^10^.

### Analysis of ancestry

SAMtools *mpileup* was used to find SNPs from the RNASeq data using the 1000 Genomes dataset as reference. Using QuASAR-vcr^44^, each sample was genotyped and a set of SNPs common to all samples was determined using bcftools. These genotypes were converted to nominal variables and R was used to perform PCA analysis on samples using SNPs, and loadings were used subsequently as a race (genotype) covariate. The 1000 Genomes subjects were included in a separate test as reference.

### Differential expression analysis

DESeq2^10^ was used to identify differentially expressed genes in drug abusers. Although demographics and sample quality did not differ significantly between the opioid abuse and control groups (Table 1), variances due to covariates of age, brain pH, RIN, and ancestry (Supplementary Fig. S1) were included in the linear regression model during statistical testing. As the mean of the genotypes were not removed, the first PC actually captures overall allele frequencies but not ancestry-related information. In contrast, PC2 and PC3 (Supplementary Fig. S1) capture continental ancestry axes of variation as also demonstrated by recorded ethnicity of subjects. In terms of drugs, the further addition of cocaine (present in a subgroup of opioid abusers) as a covariate did not significantly impact the list of differentially expressed genes (~2% difference). A pre-filtering step was used to remove low count (<1) genes. Independent Hypothesis Weighting was used for calculating adjusted p-values, with p-adj < 0.1 considered differentially expressed. Variance stabilizing transformation was performed on raw read counts as a means of normalization for downstream analysis.

### WGCNA, functional annotation, and receiver operating characteristic curve analyses

Weighted correlation network analysis (WGCNA)^11^ was used to construct a co-expression network in an attempt to identify modules (clusters) of highly correlated genes associated with opioid abuse. Variance stabilized read counts were used as input for WGCNA. As was done with DESeq2, the influence of covariates was regressed out prior to WGCNA analysis. Low abundant transcripts (with a DESeq2 *baseMean* value < 1) were removed. The WGCNA function, *blockwiseModules*, was used with the following parameters: power = 16, networkType = “signed”, *corType = *“bicor”, TOMType = “signed”, *minClusterSize = *5, mergeCutHeight = 0.25, and maxBlockSize = 40000. Briefly, the function constructs correlation matrices based on similarity in the expression profiles of genes in the dataset. These correlation matrices are then raised to a soft-thresholding power, *B*, converting them to measures of adjacency, which are then used to calculate the topological overlap dissimilarity measures between genes. Using the WGCNA function, *pickSoftThreshold*, we determined that a *B* value of 16 would satisfy the scale-free topology criterion. Using hierarchical clustering, modules consisting of co-expressed genes are then detected based on TOM values and similar modules are subsequently merged. With a merge cut height of 0.25, WGCNA identified a final list of 24 distinct modules of putatively co-expressed genes potentially representing broad biological functions (plus a grey module of genes that did not segregate into other modules).

In order to isolate specific modules that may be biologically relevant to opioid abuse, a Pearson correlation between the module eigengene (ME) of each module and the phenotype (opioid abuse) was carried out. The ME is the first principle component of a module that essentially serves as a representation of the gene expression patterns within a module. Furthermore, module hub genes (defined as module membership ≥ 0.75 and gene significance ≥ 0.3) were identified. David Gene Ontology (GO) analysis^12^ was used to assign functional annotations (e.g. biological processes) and to determine transcription factor binding site enrichment for the 4 largest modules significantly correlated with opioid abuse (plum4 was excluded because of its limited number of genes). All genes included in the WGCNA dataset were used as “background” during analysis. WGCNA-derived estimates of brain cell-type enrichment were identified using UserListEnrichment code^11^.

Receiver operating characteristic (ROC) curve analysis^13^ was performed (using IBM SPSS Statistics version 24) as previously described^29^. Using data from all 50 cases (Supplementary Table S1), ROC AUC and p values were calculated for each differentially expressed gene after controlling for covariates (Fig. 3, Supplementary Table S2).

### ASO knockdown of lncRNA in DAergic cell line

A clonal cell line with some properties of human DA neurons (SKNAS-G cells) was cultured and assayed as previously described^20^. Pilot experiments were used to optimize cell plating density, ASO sequence and treatments for target knockdown, and primer selection for target amplicon quantification. In final experiments, 400,000 cells were added to 6-well plates, followed after 1 h by treatment with 1 nM phosphorothioate-stabilized 20mer antisense oligonucleotide (ASO; Integrated DNA Technologies, USA; sequences in Supplementary Table S4) directed against targeted lncRNAs or a scrambled negative control ASO. Cells were harvested 24 h after plating, with RNA purification and quantification as described for tissue samples. RNA was then reverse-transcribed using SuperScript™ II Reverse Transcriptase, and RT-qPCR conducted with a StepOne™ Real-Time PCR System using reaction mixtures that consisted of the Applied Biosystems SYBR Green PCR Master Mix, gene-specific forward and reverse primers (Supplementary Table S4), and diluted cDNA samples Two independent experiments (with N = 4–5 independent samples per group per experiment) were conducted for each ASO. Independent samples t-tests (calculated using IBM SPSS Statistics version 24) identified significant differences in each experiment which were then pooled for purposes of presentation and final statistical analysis. Similar results were obtained whether ASO targeted and off-target effects were analyzed individually (as in Fig. 4 and Supplementary Fig. S3) or were expressed as ratios of target transcripts to actin transcript.

### *In situ* hybridization histochemistry

The cellular localization of lncRNA MIR210HG was determined using previously published methods^34,45–52^. Digoxigenin-labeled antisense or sense (control) riboprobes were transcribed from cloned DNA sequences derived using the same parameters as qPCR validation experiments (Supplementary Table S4), and signal developed using anti-digoxigenin-alkaline phosphatase conjugated Fab fragment with NBT/BCIP as substrate. Images were captured using an Olympus BX53 microscope and 60X immersion objective and CellSens software.

## Supplementary information

Supplementary Table S2

Supplementary Table S3

Supplementary Information

## Data Availability

All raw sequencing files have been submitted to the NCBI Sequence Read Archive (SRA; www.ncbi.nlm.nih.gov/sra) under accession number PRJNA492904. Each sample has an individual BAM file matched to a biosample metadata chart stating the details of the experiment associated with that sample. Each sample has its own unique identifier in the chart as well as demographics (e.g. sex and race) and drug status, matching the data found in manuscript Supplementary Table 1. Information regarding the experiment is available through the bioproject link found under the SRA accession number.
