# Supplementary material for: Differentially expressed gene networks, biomarkers, long noncoding RNAs, and shared responses with cocaine identified in the midbrains of human opioid abusers
Source: Sci Rep. 2019 Feb 7;9:1534. doi: 10.1038/s41598-018-38209-8 (PMC6367337; doi:10.1038/s41598-018-38209-8)
Supplement: Supplementary file 3 — Supplementary Information [file 41598_2018_38209_MOESM3_ESM.pdf]

Differentially expressed gene networks, biomarkers, long noncoding RNAs, and shared responses with cocaine identified in the midbrains of human opioid abusers

Manal H. Saad, Matthew Rumschlag, Michael H. Guerra, Candace L. Savonen, Alaina M. Jaster, Philip D. Olson, Adnan Alazizi, Francesca Luca, Roger Pique-Regi, Carl J. Schmidt, Michael J. Bannon

LIST OF SUPPLEMENTARY TABLES & FIGURES

Supplementary Table S1. Detailed characteristics of study subjects and specimens.

Supplementary Table S2. Complete list of differentially expressed genes, WGCNA modular assignments, biotypes, ROC AUC values, and overlap with genes previously found differentially expressed in cocaine abusers.

Supplementary Table S3. RNA-seq reads for each subject after removal of PCR duplicates and after HTSeq gene alignment.

Supplementary Table S4. Oligonucleotides used for RT-qPCR analysis and ASO knockdown experiments. Sequences listed 5'-3'. Amplicon size for PCR products is indicated. For ASO oligonucleotides, nucleotide modifications are indicated. m, methyl; * phosphothiolate bond.

Supplementary Figure S1. Covariate variance partitioning. Violin plots show variance accounted for by genetics (i.e. ethnicity, race: PC2 & PC3), subject age, and sample quality (brain pH, RIN).

Supplementary Figure S2. WGCNA clustering of module eigengenes. A. WGCNA gene clustering dendrogram showing hierarchical clustering of modules by measures of dissimilarity based on topological overlap. Rows correspond to individual modules. B. Dendrogram of WGCNA module eigengenes, with a cut height of 0.25 (red line), which merged similar modules with a correlation of ≥0.75, resulting in 25 distinct modules.

Supplementary Figure S3. Boxplots of data for the top 10 up-regulated, ROC-significant genes shown in Fig. 3. Median values, upper and lower quartiles, and maximum and minimum values shown for each gene.

Supplementary Figure S4. LncRNA *LINC00963* regulates expression of downstream target gene *GPT2*. A. The relationship between gene significance (a measure of the correlation between a given gene and the opioid variable) and module membership (the relationship between a given gene and the module eigengene) for genes in the firebrick3 module. Overall correlation and significance noted at the top. Highly correlated genes profiled in B are indicated. B. DAergic SNKAS-G cells were treated acutely with ASO directed against *LINC00963* or a negative control ASO. Selective knockdown of *LINC00963* resulted in corresponding, selective reductions in *GPT2* but not *ACTB* expression. The results of two independent experiments were combined (total N=9 samples per group). *p<0.000005.

Supplementary Figure S5. LncRNA *MIR210HG* is expressed in human ventral midbrain DA neurons. A. *MIR210HG* transcript was visualized by in situ hybridization histochemistry, with alkaline phosphatase substrate NBT/BCIP seen as a blue precipitate in a DA neuron, also identifiable by the presence of endogenous neuromelanin (brown). B. *SLC6A3* transcript, with its exquisite DA cell-specificity of expression, was visualized as a positive control for specificity of DA cell labeling. C. *PY2R12* transcript, a known marker for human microglia, served as a negative control for DA cell labeling; as expected, robust *PY2R12* expression was seen in numerous small microglia adjacent to an unlabelled DA neuron.

Supplementary Figure S6. WGCNA-derived estimates of brain cell enrichment for the major opioid-responsive modules. Cell type-associated terms, identified using WGCNA UserListEnrichment code, are listed in descending order of statistical significance (Y axis) for each module. References cited correspond to manuscript refs. 46-52.

Supplementary Table S1. Characteristics of study subjects and specimens

Control Subjects (n=20)

Case	Cause of Death	Age	Race/Sex	Brain pH	RIN	Opioids	Cocaine
**1**	MGSW	41	BM	6.64	8.04	Neg	Neg
**2**	Aortic dissection, HTCVD	42	BM	6.78	7.03	Neg	Neg
**3**	ASCVD	46	BM	6.71	7.20	Neg	Neg
**4**	ASCVD	47	BM	6.5	7.17	Neg	Neg
**5**	HTCVD	47	BM	6.6	7.60	Neg	Neg
**6**	GSW	49	BM	6.67	6.70	Neg	Neg
**7**	MGSW	49	BM	6.62	7.70	Neg	Neg
**8**	MGSW	50	BM	6.71	7.70	Neg	Neg
**9**	HTCVD	50	BM	6.27	7.10	Neg	Neg
**10**	ASCVD	51	WM	6.68	7.67	Neg	Neg
**11**	Cardiomyopathy	51	WM	6.57	7.60	Neg	Neg
**12**	ASCVD	52	WM	6.58	7.37	Neg	Neg
**13**	ASCVD	52	BM	6.71	6.50	Neg	Neg
**14**	HTCVD	54	WM	6.43	7.90	Neg	Neg
**15**	HTCVD	54	BM	6.58	7.00	Neg	Neg
**16**	ASCVD	54	BM	6.43	7.40	Neg	Neg
**17**	Cardiac dysrhythmia, myocarditis	54	BM	6.53	7.70	Neg	Neg
**18**	ASCVD	54	BM	6.72	8.30	Neg	Neg
**19**	ASCVD	55	WM	6.73	7.70	Neg	Neg
**20**	ASCVD	56	WM	6.57	6.37	Neg	Neg
	**Mean ±**	50	70% BM	6.60	7.39		
	**SEM**	0.93		0.03	0.11		

Drug Abuse Subjects (n=30)

Case	Cause of Death	Age	Race/Sex	Brain pH	RIN	Opioids	Cocaine
**21**	Drug abuse	40	WM	6.49	6.80	Fe	Pos
**22**	Drug abuse	40	WM	6.27	6.20	Mo	Neg
**23**	Drug abuse	41	BM	6.6	7.90	Fe, He	Neg
**24**	Drug abuse	44	BM	6.76	6.80	Co, He, Mo	Pos
**25**	Drug abuse	45	BM	6.52	6.73	Mo	Pos
**26**	Drug abuse	46	WM	6.81	6.70	Fe, Hy	Pos
**27**	Drug abuse	48	BM	6.46	7.33	Me	Pos
**28**	Drug abuse	49	BM	6.65	7.83	Fe	Pos
**29**	Drug abuse	50	WM	6.63	7.47	Co, Fe, He, Mo	Pos
**30**	Drug abuse	50	BM	6.47	7.77	Co, He, Mo	Pos
**31**	Drug abuse	50	BM	6.82	7.47	He, Mo	Neg
**32**	Drug abuse	50	BM	6.41	6.80	Co, He, Mo	Neg
**33**	Drug abuse	51	BM	6.55	7.40	Co, He, Mo	Pos
**34**	Drug abuse	51	BM	6.73	7.30	Co, He, Mo	Pos
**35**	Drug abuse	52	WM	6.43	7.47	Ox	Neg
**36**	Drug abuse	52	BM	6.58	7.90	Hy	Neg
**37**	Drug abuse	52	BM	6.42	7.10	Co, He, Mo	Neg
**38**	Drug abuse	53	WM	6.45	7.50	Hy	Pos
**39**	Drug abuse	53	BM	6.53	6.70	Co, He, Mo	Pos
**40**	Drug abuse	54	WM	6.62	7.80	Co, He, Mo	Pos
**41**	Drug abuse	54	BM	6.76	6.80	Fe	Neg
**42**	Drug abuse	55	BM	6.52	8.30	Co, He, Mo	Pos
**43**	Drug abuse	56	BM	6.5	7.30	He, Mo	Pos
**44**	Drug abuse	56	BM	6.49	7.97	Co, Mo	Neg
**45**	Drug abuse	56	BM	6.37	7.40	Co, He, Mo	Neg
**46**	Drug abuse	57	BM	6.47	7.23	Mo	Pos
**47**	Drug abuse	59	WM	6.43	6.70	Co, Mo	Neg
**48**	Drug abuse	59	BM	6.35	6.90	Co, Mo	Neg
**49**	Drug abuse	60	BM	6.64	6.90	Co, Mo	Pos
**50**	Drug abuse	61	BM	6.26	7.00	Fe, Mo	Pos
	**Mean ±**	51	73% BM	6.53	7.25		
	**SEM**	1.03		0.03	0.09		

Cause of death abbreviations: ASCVD, atherosclerotic cardiovascular disease; GSW, gunshot wound; HTCVD, hypertensive cardiovascular disease; MGSW, multiple gunshot wounds.
Opioid abbreviations: Co, codeine; Fe, fentanyl; He, heroin; Hy, hydrocodone; Me, methadone; Mo, morphine; Ox, oxycodone.
Other abbreviations: BM, black male; RIN, RNA integrity number; WM, white male.

Supplementary Table S4. Oligonucleotide sequences used for ASO and RT-qPCR experiments

**ASO Sequences**		
LINC00963		mU*mU*mU*mC*mC*A*T*C*G*A*T*G*G*C*C*mU*mG*mC*mA*mU
MIR210HG		mA*mG*mU*mC*mC*C*C*A*G*A*G*G*T*G*G*mC*mC*mC*mU*mC
Control		mG*mC*mG*mA*mC*T*A*T*A*C*G*C*G*C*A*mA*mU*mA*mU*mG*
**Primer sequences for RT-qPCR validation**		
ACTB 155BP	Forward Primer	ACT GGA ACG GTG AAG GTG AC
	Reverse Primer	GCT TTT AGG ATG GCA AGG GAC
GADD45B 119BP	Forward Primer	AGA CTT GGT TGA ACT TGG TTG
	Reverse Primer	TCT ATA ATT CGC AAA CTG GGA G
GPT2 177BP	Forward Primer	TGG GAA GTT AGG GAA GCT CA
	Reverse Primer	ATG TCG GTG GGT AAG GAC AG
LINC00963 93BP	Forward Primer	GCC CAG CCT GAG TCA TTT TG
	Reverse Primer	GCA TCC TCC TTC CAT CTC CAG
MIR210HG 204BP	Forward Primer	GGT CAT ATC TTC AGC CAA CAG G
	Reverse Primer	CAC GAC CCG GTC CTG ATT
NFKBIA 104BP	Forward Primer	TTC AGA TGC TGC CAG AGA G
	Reverse Primer	TCC AAA CAC ACA GTC ATC ATA G

All sequences are shown from 5’ to 3’; amplicon size for PCR products is indicated.
For ASO sequences: m indicates a 2’ O-Methyl RNA base; an asterisk (*) represents a phosphorothioate bond.

Figure S1

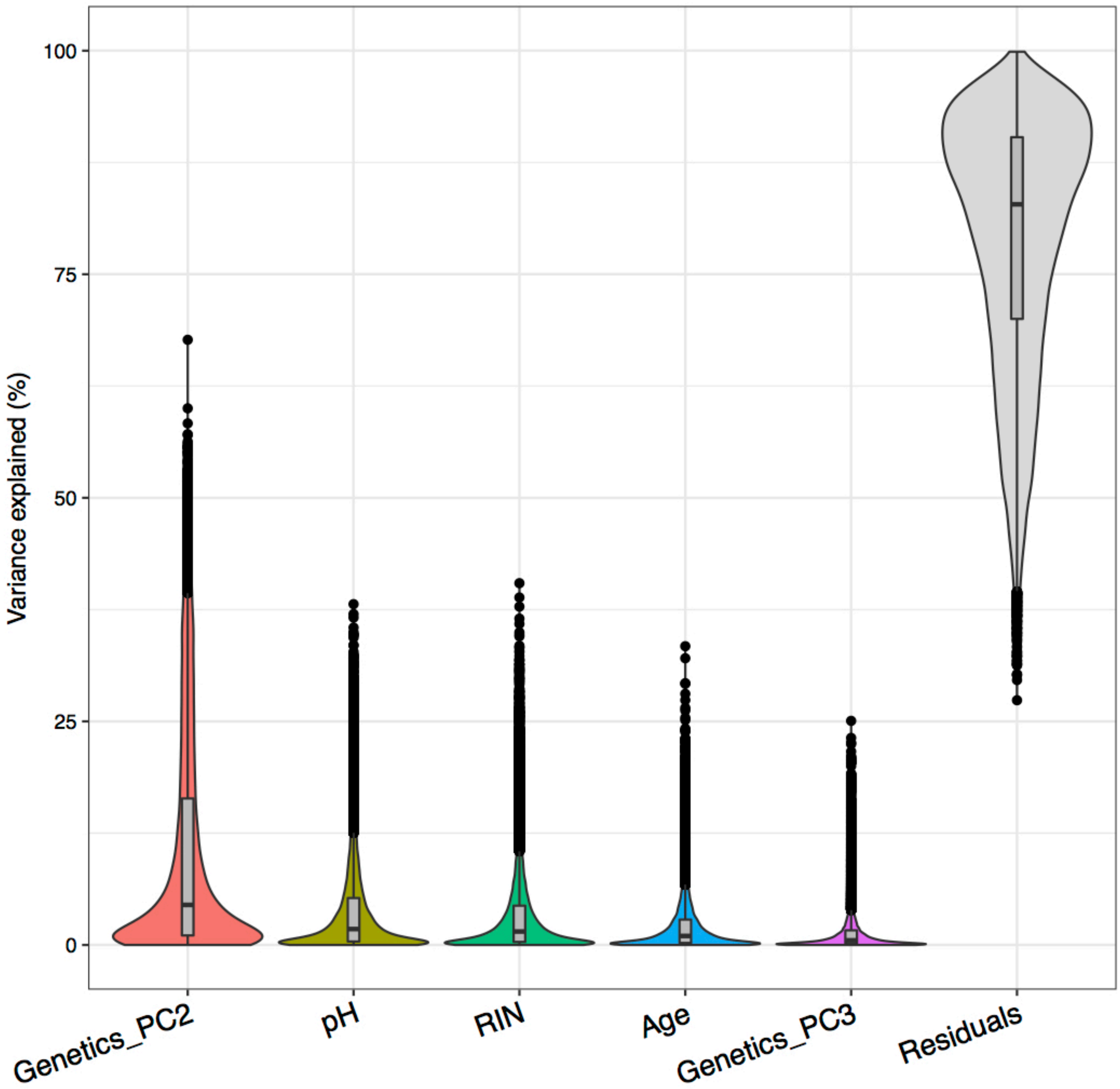

100
75
50
25
0
Variance explained (%)
Genetics_PC2
pH
RIN
Age
Genetics_PC3
Residuals

Figure S2

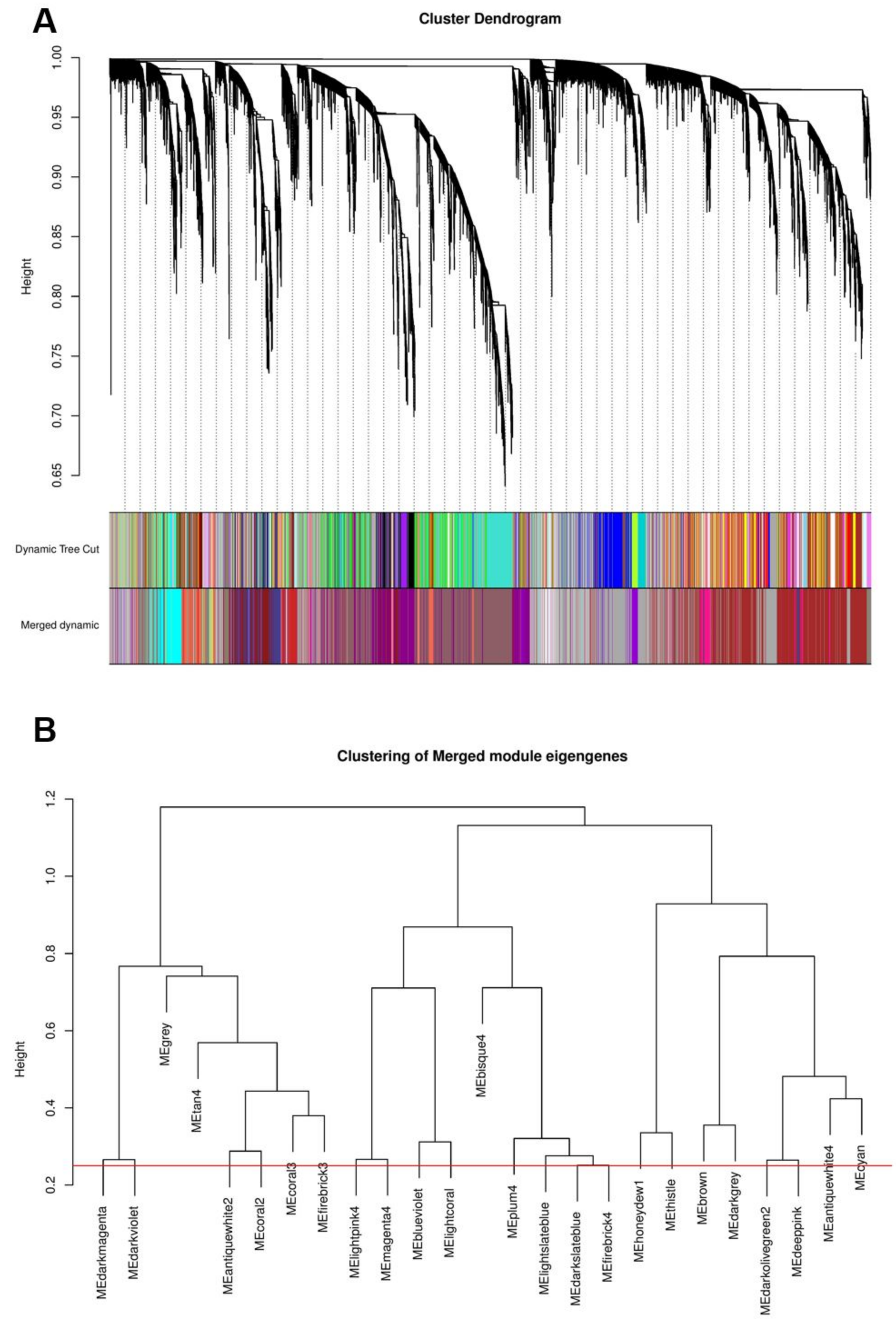

A
Cluster Dendrogram
Height
1.00
0.95
0.90
0.85
0.80
0.75
0.70
0.65
Dynamic Tree Cut
Merged dynamic
B
Clustering of Merged module eigengenes
Height
1.2
1.0
0.8
0.6
0.4
0.2
MEgrey
MEtan4
MEbisque4
MEdarkmagenta
MEdarkviolet
MEantiquewhite2
MEcoral2
MEcoral3
MEfirebrick3
MElightpink4
MEmagenta4
MEblueviolet
MElightcoral
MEplum4
MElightslateblue
MEdarkslateblue
MEfirebrick4
MEhoneydew1
MEthistle
MEbrown
MEdarkgrey
MEdarkolivegreen2
MEdeeppink
MEantiquewhite4
MEcyan

Figure S3

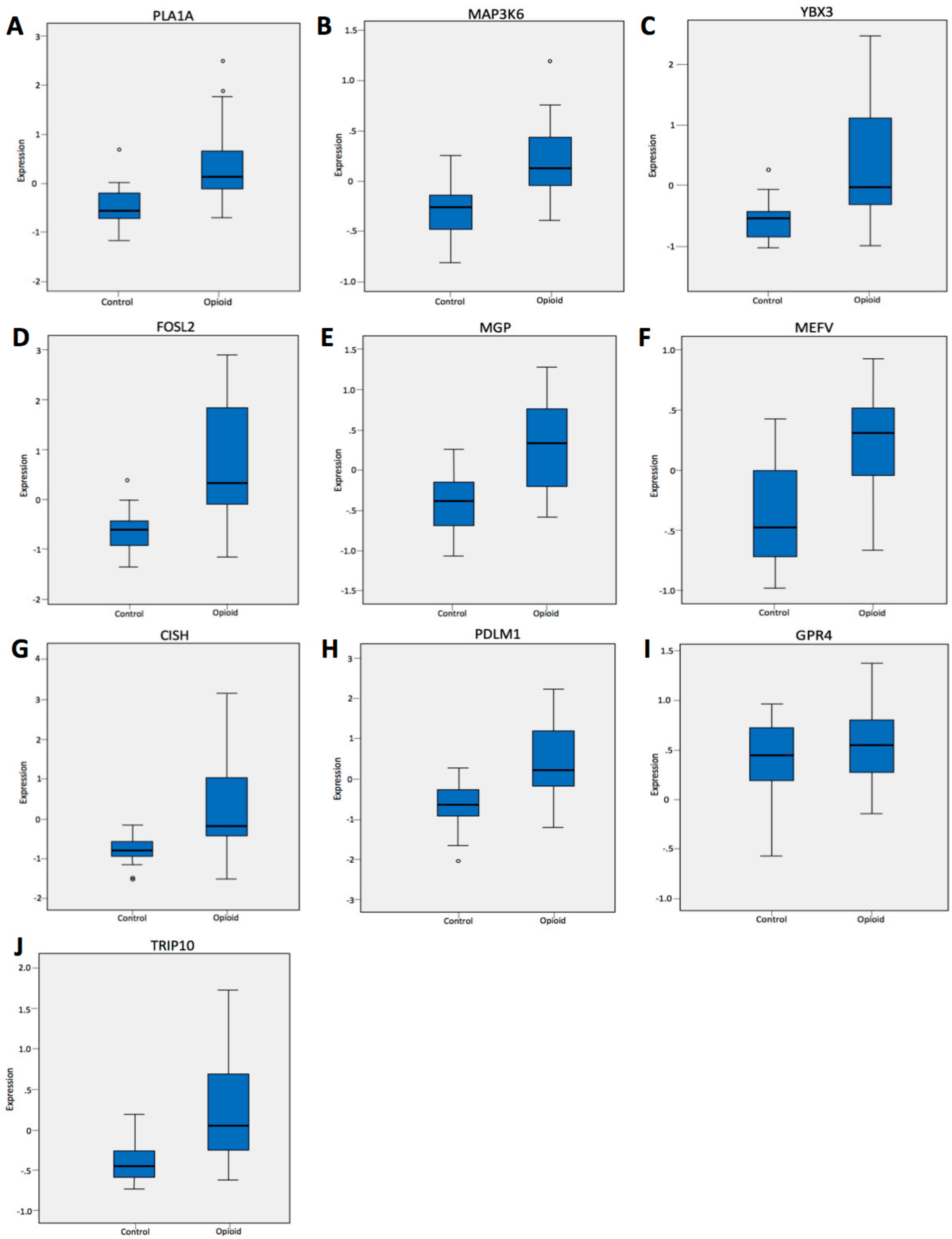
A
PLA1A
B
MAP3K6
C
YBX3
D
FOSL2
E
MGP
F
MEFV
G
CISH
H
PDLM1
I
GPR4
J
TRIP10
Expression
Control
Opioid

Figure S4

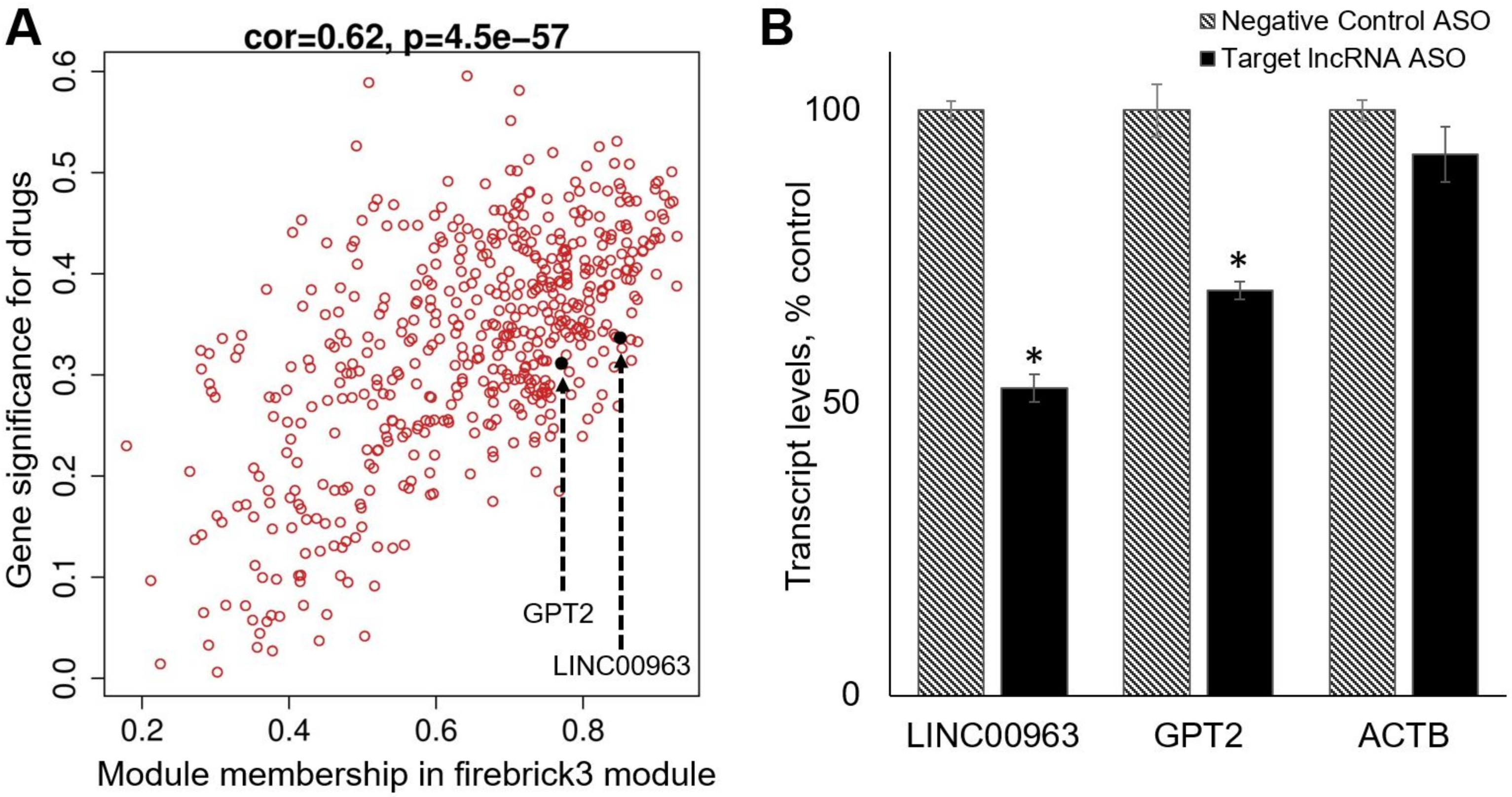
A
cor=0.62, p=4.5e−57
Gene significance for drugs
0.0
0.1
0.2
0.3
0.4
0.5
0.6
Module membership in firebrick3 module
0.2
0.4
0.6
0.8
GPT2
LINC00963
B
Negative Control ASO
Target lncRNA ASO
Transcript levels, % control
0
50
100
*
*
LINC00963
GPT2
ACTB

Figure S5

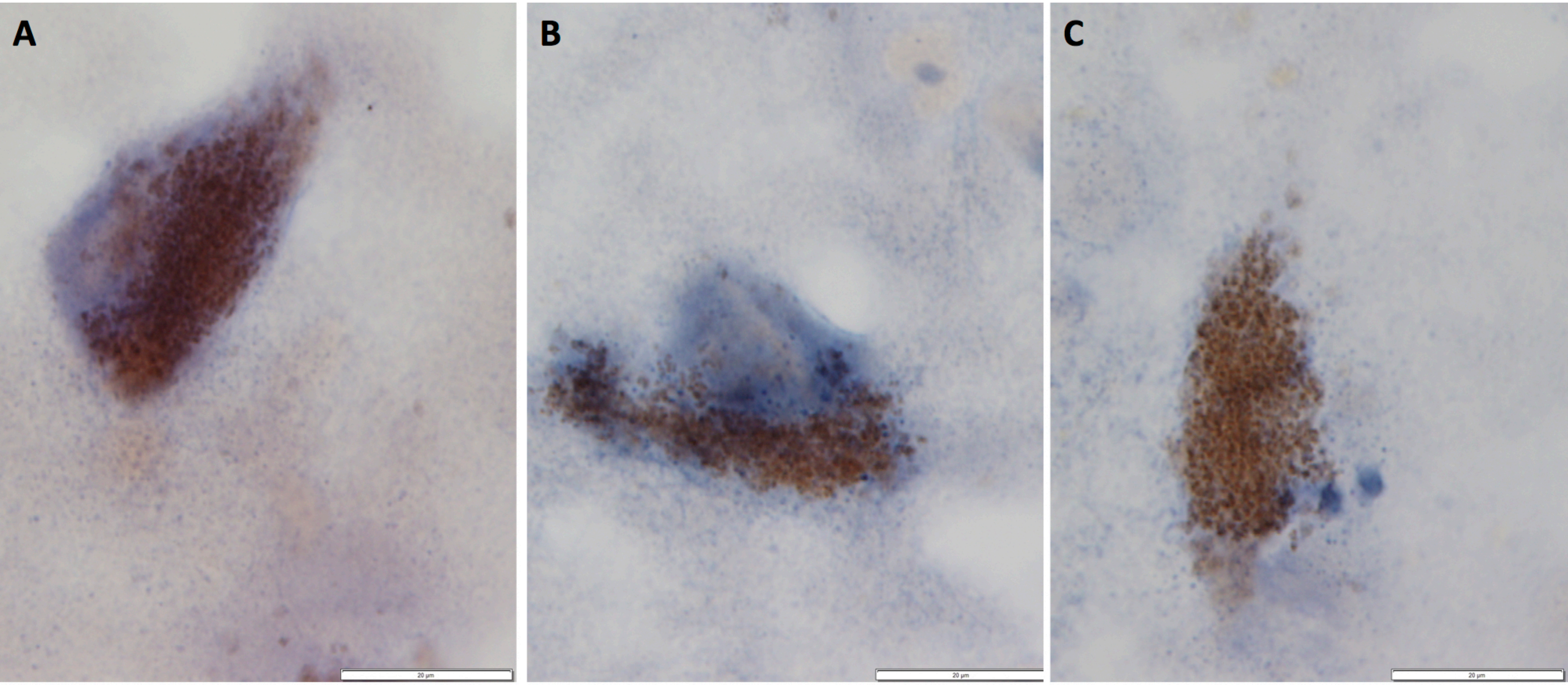

Figure S6

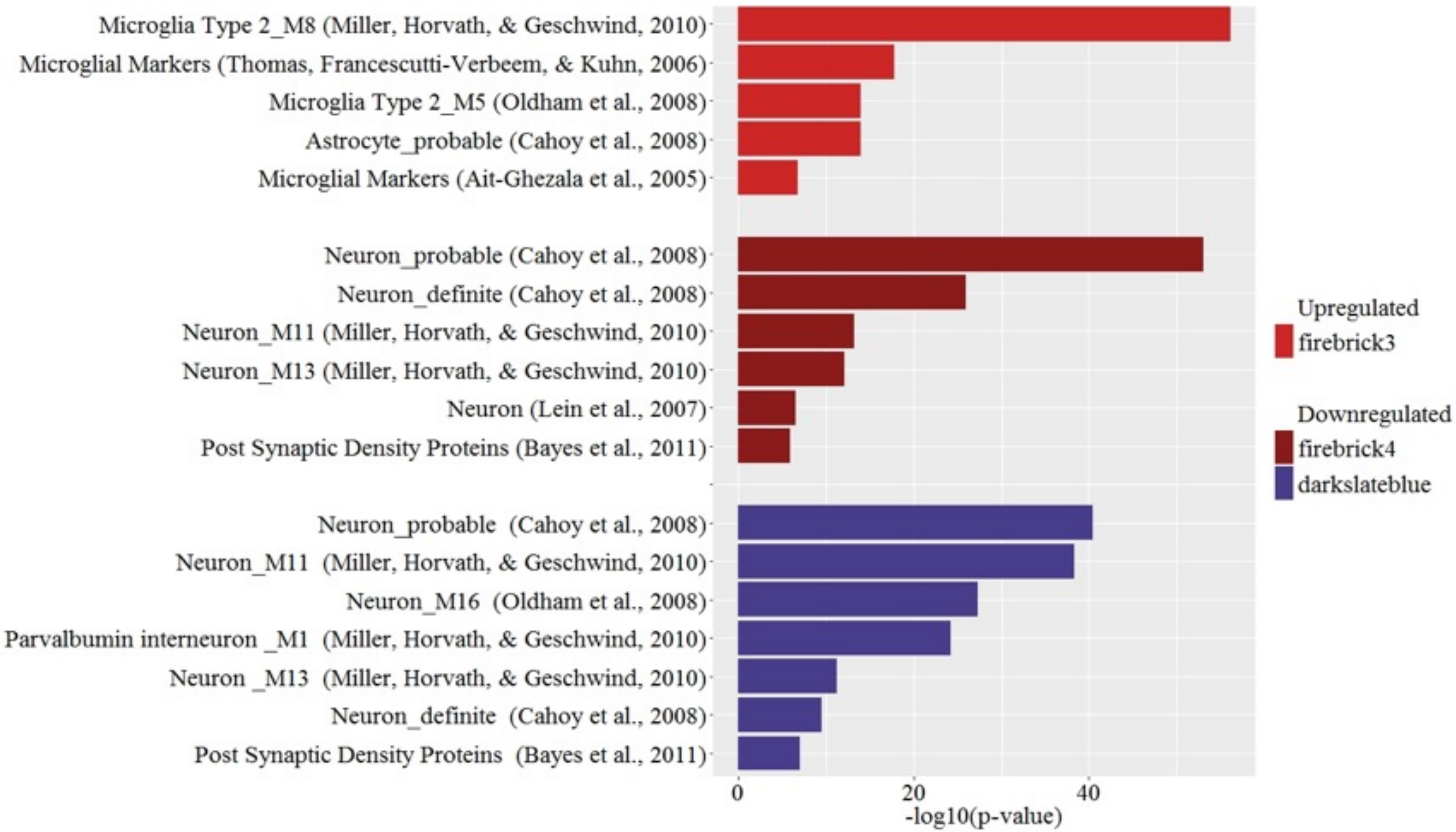
Microglia Type 2_M8 (Miller, Horvath, & Geschwind, 2010)
Microglial Markers (Thomas, Francescutti-Verbeem, & Kuhn, 2006)
Microglia Type 2_M5 (Oldham et al., 2008)
Astrocyte_probable (Cahoy et al., 2008)
Microglial Markers (Ait-Ghezala et al., 2005)
Neuron_probable (Cahoy et al., 2008)
Neuron_definite (Cahoy et al., 2008)
Neuron_M11 (Miller, Horvath, & Geschwind, 2010)
Neuron_M13 (Miller, Horvath, & Geschwind, 2010)
Neuron (Lein et al., 2007)
Post Synaptic Density Proteins (Bayes et al., 2011)
Neuron_probable (Cahoy et al., 2008)
Neuron_M11 (Miller, Horvath, & Geschwind, 2010)
Neuron_M16 (Oldham et al., 2008)
Parvalbumin interneuron _M1 (Miller, Horvath, & Geschwind, 2010)
Neuron _M13 (Miller, Horvath, & Geschwind, 2010)
Neuron_definite (Cahoy et al., 2008)
Post Synaptic Density Proteins (Bayes et al., 2011)
0
20
40
-log10(p-value)
Upregulated
firebrick3
Downregulated
firebrick4
darkslateblue
